# The hydrobioid freshwater gastropods (Caenogastropoda, Truncatelloidea) of Greece: new records, taxonomic re-assessments using DNA sequence data and an update of the IUCN Red List Categories

**DOI:** 10.3897/zookeys.350.6001

**Published:** 2013-11-14

**Authors:** Canella Radea, Aristeidis Parmakelis, Vassilis Papadogiannis, Despoina Charou, Kostas A. Triantis

**Affiliations:** 1 Faculty of Biology, Department of Ecology and Systematics, National and Kapodistrian University of Athens, 15784 Panepistimioupolis, Greece; 2 Conservation Biogeography and Macroecology Program, School of Geography and the Environment, University of Oxford, Oxford OX1 3QY, UK

**Keywords:** Hydrobioids, Rissooidea, Truncatelloidea, Gastropoda, Greece, freshwater, taxonomy, IUCN status, conservation

## Abstract

Hydrobioid freshwater gastropods were collected from mainland and insular Greece. Several threatened taxa, such as *Graecoanatolica vegorriticola*, *Pseudamnicola negropontina*, *Pseudamnicola pieperi*, *Pseudobithynia eubooensis* and *Pseudoislamia balcanica*, were recorded from new localities. *Trichonia trichonica*, which has been considered extinct from its type locality for the last twenty eight years, was re-discovered, whereas the presence of *Daphniola exigua*, *G. vegorriticola*, *Marstoniopsis graeca*, *P. pieperi* and *Pseudobithynia trichonis* in their type localities was verified. The taxonomic status of *P. negropontina* and the newly discovered populations of *G. vegorriticola* was elucidated using *COI* sequence data. The new data recorded during this survey indicate that the IUCN status of some Greek endemic hydrobioids needs to be updated.

## Introduction

Hydrobioid gastropods include the family Hydrobiidae Troschel, 1857 and several other families of Rissooidea s.l. that resemble Hydrobiidae in general features (Hershler and Ponder 1998). Rissooidea s.l. is one of the largest and most taxonomically challenging gastropod superfamilies (Bouchet and Rocroi 2005). Recently, Criscione and Ponder (2013) using molecular data showed that that there are two major clades encompassing taxa previously included in Rissooidea. These clades are the Rissooidea s.s. and the Truncatelloidea, the latter containing the hydrobioid families (Wilke et al. 2013).

Greek freshwater ecosystems are widely recognized as hotspots of European freshwater biodiversity (e.g. Glöer and Maassen 2009, Glöer et al. 2010, Oikonomou et al. 2012). Hydrobioids are in accordance with this observation since they are highly diverse; 77 species and subspecies belonging to 29 genera have already been recorded in Greek freshwater systems, most of them (i.e. 68.4% of the species and subspecies and 34.5% of the genera) being endemic for Greece (Bank 2006, Falniowski and Szarowska 2011a, Radea 2011, Szarowska and Falniowski 2011a, Glöer and Georgiev 2012, Georgiev 2013a, b, Radea et al. 2013). Nevertheless, the freshwater gastropod fauna of Greece remains understudied compared to other taxa (i.e. freshwater fishes) due to the complex hydrographic network of its drainages.

Almost all described Greek hydrobioid species are included in the IUCN Red List of Threatened Species (2012) and 55% of them have been classified as threatened. *Graecoanatolica macedonica* Radoman & Stankovic, 1978 is characterized as Extinct, and is followed by twenty four species that have been classified as Critically Endangered, five as Endangered, nine as Vulnerable, three as Near Threatened, five as Least Concern and twenty two as Data Deficient.

During 2012, several localities across Greece were sampled by the authors for hydrobioid freshwater gastropods. The sampling took place following the goals of the research project “Species on the brink of extinction” that was funded by the public benefit foundation “John S. Latsis”. The goals of this project was a) to assess and evaluate the population status of 10 freshwater snails species of Greece (9 endemics) which, according to the recent report from the International Union for Conservation of Nature (IUCN), are classified as either Extinct or Critically Endangered, b) to evaluate the status of the wetlands these species are present in and to assess the main anthropogenic regime of threats. In the network of localities sampled for the purposes of the project, we added several more localities hosting water bodies that are frequently reported in the freshwater literature of Greece or are in the vicinity of the primarily targeted localities. Therefore, we do not consider our fieldwork to be exhaustive; rather it is focused on freshwater localities that have been searched before, are frequently reported in the literature, as hosting (or having hosted) threatened species, and we complemented these localities with surrounding ones that could have served as the refuges of the threatened species.

Some of the findings of this survey as well as suggestions for the IUCN status update of some hydrobioids collected, are presented and discussed herein. Additionally, using *COI* sequence data generated from some specimens, we aimed to elucidate the taxonomic status of:

(i) The Greek endemic taxon of the genus *Pseudamnicola* Paulucci, 1878, namely *Pseudamnicola (Pseudamnicola) macrostoma negropontina* (Clessin, 1878). Based on the slight morphological and anatomical differences between *Pseudamnicola (Pseudamnicola) macrostomanegropontina* and *Pseudamnicola (Pseudamnicola) macrostoma macrostoma* (Küster, 1853), Szarowska et al. (2006) claimed that these taxa should be considered as distinct species. This claim was evaluated in the light of the generated sequence data.

(ii) Two new populations of the genus *Graecoanatolica* Radoman, 1973, which were recorded in Sterea Ellada (Voiotia). The morphological and anatomical studies were not conclusive in assigning these populations to the known Greek *Graeconatolica* species, *Graeconatolicavegorriticola* (Schütt, 1962) and *Graecoanatolica macedonica*, or even to a new species of *Graecoanatolica*. Therefore, *COI* sequence data were used to compare the *Graecoanatolica vegorriticola* specimens collected from the type locality of the species, with those of the newly located southern populations.

Overall, the current study offers new distributional data and, in the light of these, is evaluating the conservation status of some hydrobioid species of Greece. Furthermore, using newly generated *COI* sequence data, we are resolving taxonomic uncertainties that could not be elucidated based on morphology alone.

## Methods

**Figure 1.**
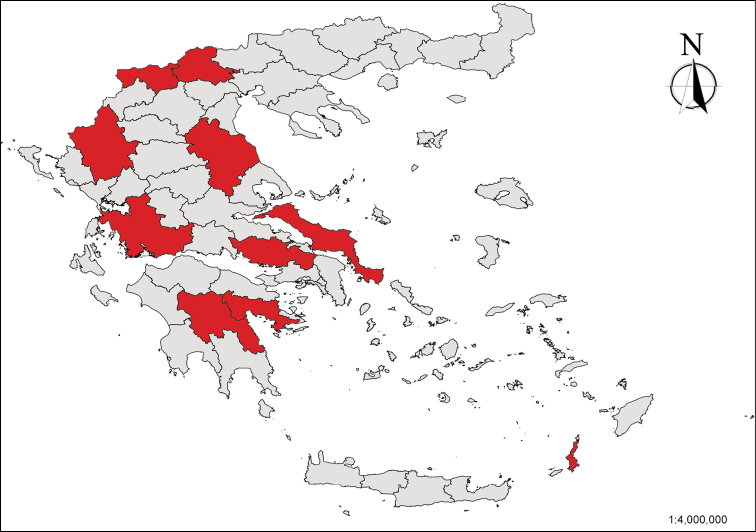
Map showing the administrative units of Greece where “hydrobioid” localities were sampled during the fieldwork of this study.

**Figure 2.**
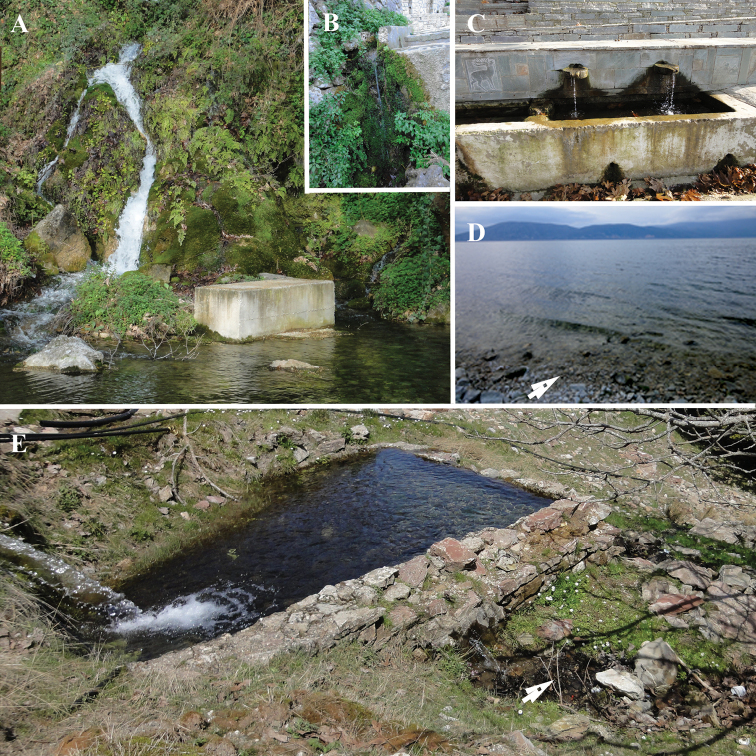
Sampling localities. **A** Ag. Sophia, Aitoloakarnania **B** Olympos, Karpathos **C** Stoupaioi, Evvoia **D** Peraia, Lake Vegorritis **E** Megali Vrysi, Argolida. The arrows point the exact site where the specimens were found.

The freshwater localities of almost all the threatened hydrobioids of Greece were carefully sampled (Fig. 1). The snails were hand collected from stones, gravel and dead leaves (Fig. 2). Specimens were placed into vials filled with water and were transported alive to the laboratory. A portion of the specimens collected from each locality was stored in -20°C for molecular analysis, whereas others were preserved un-relaxed in 70% ethanol for morphological and anatomical studies.

Shell characters (shell height and width, apertural height and width) were measured using the micrometer of a stereomicroscope (Stemi 2000-C, Zeiss). Digital pictures were taken using a Canon EOS 1000D camera that was attached to the stereomicroscope. During this procedure, the specimens were submerged into water in order to avoid the malformation of important taxonomic features that can be caused by the long-term tissue preservation buffers.

Details of the specimens used in the molecular analyses are provided in Table 1. The entire individuals were used for total genomic DNA isolation. In total, we extracted DNA from one specimen of *Pseudamnicola macrostoma macrostoma* and sixteen specimens of *Graecoanatolica* from Pella and Voiotia.

**Table 1.** Details on the origin of the specimens used in the *COI* sequence divergence analyses.

Species	Number of specimens	Nomos	Exact locality	Habitat	Latitude, Longitude	Sample code	*COI* Accession Numbers
*Graecoanatolica vegorriticola*	6	Voiotia	Orchomenos, Pigi Chariton	Spring	38°29'41"N, 22°58'23"E	GraVeg_Cha	KF758767-72
*Graecoanatolica vegorriticola*	2	Voiotia	Livadia, Spring of Krya	Spring	38°25'49"N, 22°52'22"E	GraVeg_Kr1	KF758773-74
*Graecoanatolica vegorriticola*	8	Pella	Pella, Vegorritis Lake	Lake	40°44'38"N, 21°49'07"E	GraVeg_Veg	KF758775-82
*Pseudamnicola macrostoma macrostoma*	1	Attiki	Marathon, Kato Souli	Stream	38°09'28"N, 24°00'19"E	PsdMac_KaSou	KF758783
*Pseudamnicola macrostoma negropontina*	1	Evvoia	Marmaris	-		-	EF061915 (Szarowska et al. 2006) †

† According to the GenBank registration records the locality of origin of EF061915 is Kato Souli. However, this must be an erroneous record since in the reference (Szarowska et al. 2006) associated with this accession number, it is clearly stated that the specimen of *Pseudamnicola macrostoma negropontina* originated from Marmaris (Marmari) in Evvoia Island.

DNA was extracted using the Purelink Genomic DNA mini kit (Invitrogen) following the manufacturers’ protocol. A fragment of the mitochondrial gene cytochrome oxidase subunit I (*COI*) was amplified from each specimen using the universal primers LCO1490 and HCO2198 (Folmer et al. 1994). PCR was performed in a 25 μl volume, in which 1–2 μl of template DNA was mixed with 0.2 mM dNTPs, 0.4 mM of each primer and 0.5 units of Taq polymerase. The concentration of the MgCl_2_ was 3.5 mM. Thermocycling was performed in a MyCycler (Biorad) thermocycler. The cycle program comprised an initial denaturation step at 95 °C for 3 min, followed by 40 cycles of 15 s at 95 °C, 1 min at 42 °C, and 1.5 min at 72°C. The cycling was ended with 10 min sequence extension at 72 °C. Automated sequencing of both strands of the PCR amplicons was performed in a PE-ABI3740 automated sequencer (using Big-Dye terminator chemistry).

The primers in the sequencing reactions were the same as in the PCR amplifications. Sequences generated for this study were deposited in GenBank under the accession numbers provided in Table 1.

### Sequence alignment and genetic data analysis

The newly generated sequences were viewed and edited using CodonCode Aligner v. 2.06 (Genecodes Corporation). The authenticity of the mtDNA sequences and the homology with the targeted mitochondrial gene were evaluated by a BLAST search in the NCBI genetic database (http://blast.ncbi.nlm.nih.gov/Blast.cgi). All sequences alignments were performed with CodonCode Aligner v. 2.06 by implementing the Clustal algorithm.

For deciphering whether the two *Pseudamnicola* taxa represent two different species or if they are subspecies of a single species, we estimated the sequence divergence separating them by using the Kimura 2-parameter (K-2p) model (Kimura 1980) of nucleotide substitution as implemented in MEGA5 (Tamura et al. 2011). The estimated *COI* sequence divergence of the two *Pseudamnicola* taxa (one produced for this study and one retrieved from GenBank, see Table 1) was compared to that separating well defined *Pseudamnicola* species. The sequence data for these species were also retrieved from GenBank.

In the case of the newly discovered *Graeconatolica* populations from southern Greece, based on the K-2p model, we estimated the *COI* sequence divergence of these specimens from the *Graecoanatolica vegorriticola* specimens sampled from the type locality of the species.

An alphabetical sequence of families, genera and species applies to the hydrobioid list below. Abbreviations used: Nom.= Nomos (administrative unit).

## Results

### Family Amnicolidae Tryon, 1863

#### Genus *Marstoniopsis* van Regteren Altena, 1936

##### Marstoniopsis graeca

(Radoman, 1978)

http://species-id.net/wiki/Marstoniopsis_graeca

###### New records.

Nom. PELLAS: Lake Vegorritis, stony bank close to Peraia, ca 515 m asl, 40°44'38"N, 21°49'07"E, 15.xi.2012, Radea and Parmakelis.

###### Remarks.

*Marstoniopsis graeca* was described from Lake Vegorritis as *Parabythinella graeca* Radoman, 1978 and, according to Radoman (1983), the stony east bank of the lake, to the north of the village Farangi, is the type locality of this taxon. Szarowska (2006) refers thatgross anatomy andhistology of the two taxa of *Parabythinella*, *Parabythinella graeca* and *Parabythinella macedonica* Hadžišče, 1963, do not differ from the one described for *Marstoniopsis*. The two taxa are most probably no more than two subspecies of *Marstoniopsis macedonica*. *Marstoniopsis graeca* is known only from Lake Vegorritis and Schütt (1985) considers that it is a common taxon for the area. Szarowska (2006) collected numerous specimens from Lake Vegorritis during the period 2003–2005. During our study, we found only one individual north of the locality where the species was discovered by Radoman (1978).

### Family Bythinellidae Kobelt, 1878

#### Genus *Bythinella* Moquin-Tandon, 1856

##### Bythinella cf. charpentieri

(Roth, 1855)

###### New records.

Nom. EVVOIAS: pool with *Nasturtium* sp. and *Platanus orientalis*, 3.8 km NE of Paradeisi to Ag. Dimitrios, ca 335 m asl, 38°05'26"N, 24°23'57"E, 25.xi.2012, Radea and Constantinidis; spring close to the road towards Ag. Dimitrios beach, ca 120 m asl, 38°08'26"N, 24°27'01"E, 25.xi.2012, Radea and Constantinidis; spring close to the road from Ag. Dimitrios to Kalianoi, ca 295 m asl, 38°07'21"N, 24°26'30"E, 25.xi.2012, Radea and Constantinidis; cistern, ca 0.7 km NNW of Kalianoi, ca 205 m asl, 38°07'22"N, 24°29'26"E, 26.xi.2012, Radea and Constantinidis; spring in Myloi village, ca 205 m asl, 38°01'56"N, 24°26'09"E, 26.xi.2012, Radea and Constantinidis; spring close to Stoupaioi, ca 255 m asl, 38°07'21"N, 24°18'51"E, 26.xi.2012, Radea and Constantinidis; stream with *Nasturtium* sp. and *Helosciadium* sp., on coastal flats 3.2 km NW of Marmari, ca 0 m asl, 38°04'16"N, 24°18'06"E, 26.xi.2012, Radea and Constantinidis.

Nom. VOIOTIAS: Krya spring in Livadia, ca 240 m asl, 38°25'49"N, 22°52'22"E, 17.iv.2012, Radea and Constantinidis; spring Pigi Chariton close to Orchomenos, ca 170 m asl, 38°29'41"N, 22°58'23"E, 17.iv.2012, Radea and Constantinidis.

###### Remarks.

The populations of *Bythinella* found in Evvoia and Voiotia have morphological and anatomical similarity to *Bythinella charpentieri*, which is the only known species of the genus inhabiting Attiki, Evvoia and Parnassos Mt. (Falniowski and Szarowska 2011b). In particular, the shape and the morphometry of the tubular gland of the penis, which is an important feature to distinguish *Bythinella* spp. (Glöer 2013), are identical to those of *Bythinella charpentieri*. Therefore, we consider that the populations from Evvoia and Voiotia belong to *Bythinella charpentieri*. However, molecular data would be very useful to elucidate completely their taxonomic status since Falniowski et al. (2009), Falniowski and Szarowska (2011b) and Falniowski et al. (2012) have demonstrated that there are many cryptic species in *Bythinella*, due to the morphostatic evolution, and, consequently, it is difficult to distinguish species without molecular analysis.

### Family Bithyniidae Gray, 1857

#### Genus *Pseudobithynia* Glöer & Pesic, 2006

##### Pseudobithynia euboeensis

Glöer, Falniowski & Pesic, 2010

http://species-id.net/wiki/Pseudobithynia_euboeensis

Figure 3

###### New records.

Nom. EVVOIAS: stream with dense vegetation composed mainly by *Nasturtium* sp. and *Helosciadium* sp., on coastal flats 3.2 km NW of Marmari, ca 0 m asl, 38°04'16"N, 24°18'06"E, 26.xi.2012, Radea and Constantinidis.

###### Remarks.

*Pseudobithynia euboeensis* was collected in 1985 for the first time and it was described by Glöer et al. (2010) from a damp meadow with some small water bodies, formed by the water running from a spring, at the seaside close to Marmari. The above authors report that the type locality was no longer extant in 2003 because all the water from the spring was used for irrigation.

So far, the species was known only from its type locality. The new locality is probably close to the type locality and is likely influenced by touristic activity during summer. Several specimens of *Pseudobithynia euboeensis* were found either on plant material or under stones.

**Figure 3.**
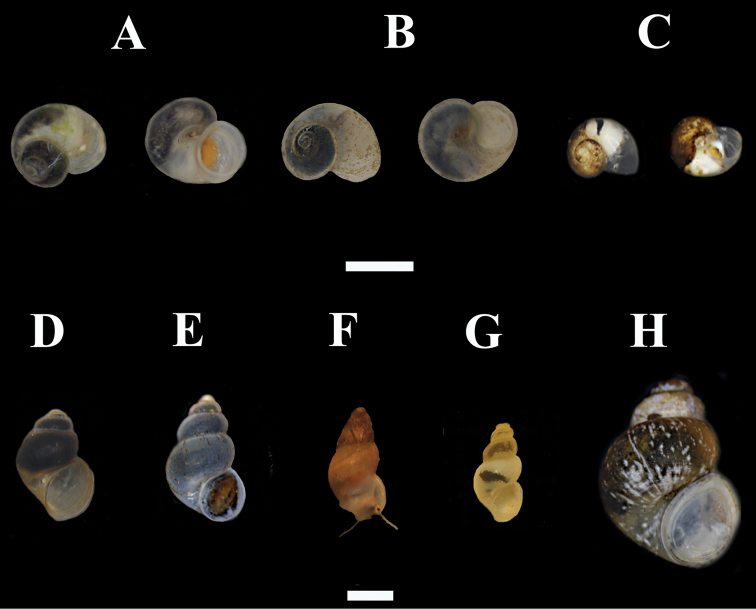
Hydrobioids collectedduring the survey in mainland and insular Greece. **A** *Daphniola exigua* (dorsal and ventral view) **B** *Isimerope semele* (dorsal and ventral view, Megali Vrysi) **C** *Pseudoislamia balcanica* (dorsal and ventral view, Ag. Sophia) **D** *Pseudamnicola pieperi* (Olympos) **E** *Radomaniola* cf. *curta* (spring of Louros river) **F** *Radomaniola* cf. *curta* (Ag. Sophia spring) **G** *Trichonia trichonica* **H** *Pseudobithynia eubooensis*. Scale bar 1 mm.

##### Pseudobithynia trichonis

Glöer, Albrecht & Wilke, 2007

http://species-id.net/wiki/Pseudobithynia_trichonis

###### New records.

Nom. AITOLOAKARNANIAS: Lake Trichonis, NE rocky and stony shore close to Loutra Myrtias, ca 15 m asl, 38°33'34"N, 21°37'33"E, 09.iii.2012, Radea, Charou, Papadogiannis, Parmakelis.

###### Remarks.

The species was described by Glöer et al. (2007) from rocks covered by microalgae, at 1m depth in the NE bank of Lake Trichonis.Our specimens were rather scattered and were found north of the locality referred by the latter authors, under stones at 10–30 cm depth. According to Albrecht et al. (2009), Lake Trichonis undergoes several human-induced environmental changes with water level loss and eutrophication being the most serious threats for gastropods living in the littoral zone.

### Family Hydrobiidae Troschel, 1857

#### *Daphniola* Radoman, 1973

##### Daphniola exigua

(A. Schmidt, 1856)

http://species-id.net/wiki/Daphniola_exigua

Figure 3

###### Remarks.

A large population of *Daphniola exigua* was found in Ag. Paraskevi spring, Tempi valley, Nom. Larissas where anthropogenic activity is high. Ag. Paraskevi spring is one of the two known localities where the species is distributed (Schütt 1980, Falniowski et al. 2007). The other locality is Daphni spring, close to Ag. Paraskevi (Radoman 1973, 1983, Falniowski et al. 2007). Radoman (1973) described the genus *Daphniola* from the latter spring, the type species being *Daphniola graeca*. Falniowski et al. (2007), using morphological and *COI* sequence data, showed that *Daphniola exigua* and *Daphniola graeca* are conspecific both belonging to *Daphniola exigua*.

#### *Graecoanatolica* Radoman, 1973

##### Graecoanatolica vegorriticola

(Schütt, 1962)

http://species-id.net/wiki/Graecoanatolica_vegorriticola

Figure 4

###### New records.

Nom. VOIOTIAS: Krya spring in Livadia, ca 240 m asl, 38°25'49"N, 22°52'22"E, 17.iv.2012, Radea and Constantinidis; spring Pigi Chariton close to Orchomenos, ca 170 m asl, 38°29'41"N, 22°58'23"E, 17.iv.2012, Radea and Constantinidis.

Nom. PELLAS: Lake Vegorritis, stony bank close to Peraia, ca 515 m asl, 40°44'38"N, 21°49'07"E, 15.xi.2012, Radea and Parmakelis.

###### Remarks.

*Graecoanatolica vegorriticola* was initially described as *Hydrobia vegorriticola* by Schütt (1962) from Lake Vegorritis and, according to Radoman (1983), the small island nearby the north bank of the lake, not far from Arnissa town, is the type locality of this species. The species was known from Lake Vegorritis and Lake Petron (Schütt 1962). Reischütz and Stummer (1990) and Hemmen and Reischütz (1996) report the presence of this species in the waterfalls of Edessa town, Central Macedonia in 1979, 1987 and 1995.

According to Schütt (1985), the abundance of *Graecoanatolica vegorriticola* used to be very high in the stony bank of Lake Vegorritis. Later on, Szarowska (2006) reported that only empty shells were found in Vegorritis in 2003. Moreover, Albrecht et al. (2006) found neither alive nor empty shells in 2005.

We found a low abundance population of *Graecoanatolica vegorriticola* on the banks of Lake Vegorritis south of Arnissa town. Furthermore, two high abundance populationsof *Graecoanatolica* cf. *vegorriticola* with many mature individuals were discovered in Livadia and Orchomenos, Central Greece, ca 270 km away from the type locality of *Graecoanatolica vegorriticola*.

*COI* sequence data generated from specimens that have been collected from Lake Vegorritis, Livadia and Orchomenos were used to elucidate the taxonomic status of the populations inhabiting the latter two localities. In total, we obtained eight *COI* sequences of *Graecoanatolica vegorriticola* specimens from Lake Vegorritis, two sequences of *Graecoanatolica* from Livadia and six sequences from Orchomenos (Table 1). The *COI* sequence divergence separating specimens from these populations and those of *Graecoanatolica vegorriticola* from Lake Vegorritis is 1.7%. The two newly discovered localities are heavily influenced by tourism and agriculture.

During the field survey, we devoted significant effort in finding and collecting alive specimens of the other Balkan species of this genus, *Graecoanatolica macedonica*, in the Greek part of Lake Dojran. Unfortunately, only empty shells (some of the specimens looking to have recently died) were retrieved.

**Figure 4.**
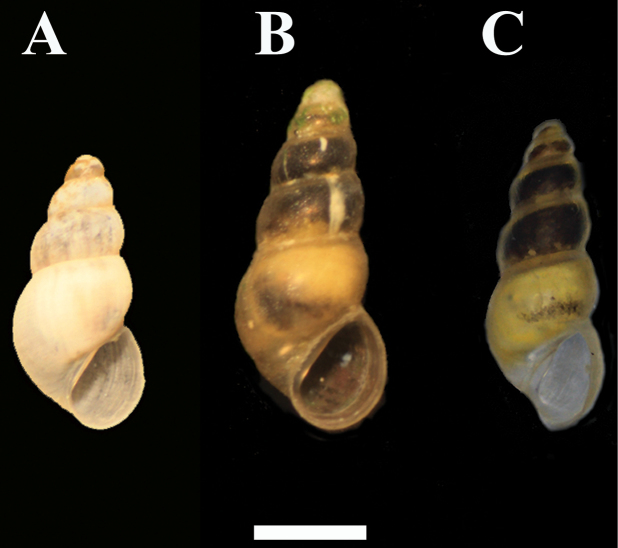
*Graecoanatolica vegorriticola* **A** Krya spring, Voiotia **B** Pigi Chariton, Voiotia **C** Lake Vegorritis, Pella. Scale bar 1 mm.

#### *Isimerope* Radea & Parmakelis, 2013

##### Isimerope semele

Radea & Parmakelis, 2013

http://species-id.net/wiki/Isimerope_semele

Figure 3

###### Remarks.

*Isimerope semele* was found in three localities of Peloponnisos, two in Nom. ARGOLIDAS (Megali Vrysi and “Second Spring”) and one in Nom. ARKADIAS (Elissonas River, Piana) (Radea et al. 2013). A fourth population of *Isimerope semele* was recorded by Falniowski and Szarowska (2011a) close to the population in Arkadia and reported by these authors as *Graecoarganiella* sp. The abundance of the known populations of *Isimerope semele* seems to be very low (Falniowski and Szarowska 2011a, Radea et al. 2013).

#### *Pseudamnicola* Paulucci, 1878

##### Pseudamnicola negropontina

(Clessin, 1878)

http://species-id.net/wiki/Pseudamnicola_negropontina

Figure 5

###### New records.

Nom. EVVOIAS: stream with dense vegetation composed mainly by *Nasturtium* sp. and *Helosciadium* sp., on coastal flats 3.2 km NW of Marmari, ca 0 m asl, 38°04'16"N, 24°18'06"E, 26.xi.2012, Radea and Constantinidis.

###### Remarks.

According to Schütt (1980) this taxon is a subspecies of *Pseudamnicola macrostoma* (Küster, 1853),i.e. *Pseudamnicola macrostoma negropontina* (Clessin, 1878),and it was described from Chalkis in Central Evvoia. Specimens of this taxon were also collected in 1985 and 2003 from an artificial pond in Marmari (Szarowska et al. 2006).

The latter authors consider *Pseudamnicola macrostoma negropontina* as a distinct species, *Pseudamnicola negropontina*. The molecular analysis that we performed after finding fresh specimens of *Pseudamnicola macrostoma* in Kato Souli, Attiki, showed that the *COI* sequence divergence separating the two taxa is 5.8% (K-2p model).

**Figure 5.**
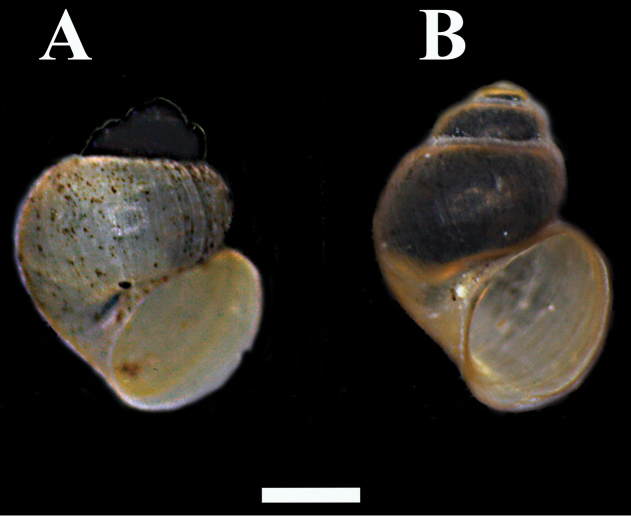
**A** *Pseudamnicola negropontina* **B** *Pseudamnicola macrostoma*. The first whorls of the shells of *Pseudamnicola negropontina* wereheavily encrusted with epibionts. Scale bar 1 mm.

##### Pseudamnicola (Pseudamnicola) pieperi

Schütt, 1980

http://species-id.net/wiki/Pseudamnicola_pieperi

Figure 3

###### New records.

KARPATHOS island: spring close to Olympos, ca 260 m asl, 35°44'00"N, 27°10'01"E, 30.xi.2012, Radea, Bazos, Contantinidis; spring close to Prasteio, ca 220 m asl, 35°43'01"N, 27°11'00"E, 01.xii.2012, Radea, Bazos, Contantinidis; spring, Vananta, ca 0 m asl, 35°46'00"N, 27°12'01"E, 02.xii.2012, Radea, Bazos, Contantinidis; spring close to Spoa, ca 295 m asl, 35°38'01"N, 27°08'01"E, 03.xii.2012, Radea, Bazos, Contantinidis; spring close to Pyles, ca 305 m asl, 35°31'01"N, 27°07'01"E, 03.xii.2012, Radea, Bazos, Contantinidis; stream crossing the secondary road leading to Ag. Nikolaos temple, ca 740 m asl, 35°34'36"N, 27°09'39"E, 15.iv.2013, Radea and Constantinidis; discharge from the pumps of a water intake built on the spring close to Ag. Nikolaos temple, ca 705 m asl, 35°34'26"N, 27°09'57"E 15.iv.2013, Radea and Constantinidis.

###### Remarks.

This species was collected for the first time by Pieper in 1977 from Aperi in Karpathos Island and described later by Schütt (1980). The type locality was the only known locality where *Pseudamnicola pieperi* occurred.

In 2012, we found several specimens of *Pseudamnicola pieperi* in the type locality. The seven new localities, where the species was found, are located in the central and northern part of the island. In the majority of the new localities, the population abundance was medium to high.

#### Genus *Pseudoislamia* Radoman, 1979

##### Pseudoislamia balcanica

Radoman, 1979

http://species-id.net/wiki/Pseudoislamia_balcanica

Figure 3

###### New records.

Nom. AITOLOAKARNANIAS: Lake Trichonis, N shore close to Dougri, ca 15 m asl, 38°36'01"N, 21°34'10"E, two specimens on leaves and stems of *Myriophyllum* sp., depth 2–4 m, 09.iii.2012, Radea, Charou, Papadogiannis, Parmakelis; spring close to Ag. Sophia, 3 km NW from Thermos, ca 305 m asl, 38°34'59"N, 21°38'56"E, three mature and some immature specimens on stones, 10.iii.2012, Radea, Charou, Papadogiannis, Parmakelis.

###### Remarks.

*Pseudoislamia balcanica* is an endemic species previously known only from its type locality, the NE rocky banks of Lake Trichonis near Myrtia (Radoman 1983). Szarowska (2006) considers this species to be extinct. In 2007 and 2008, two small populations were found near Myrtia by Albrecht et al. (2009). Our sampling revealed that although *Pseudoislamia balcanica* forms small sized populations,it is more widespread than previously thought and thrives both in lentic and lotic waters.

#### Genus *Radomaniola* Szarowska, 2006

##### Radomaniola cf. curta

(Küster, 1852)

Figure 3

###### New records.

Nom. AITOLOAKARNANIAS: Spring close to Ag. Sophia, 3 km NW from Thermos, ca 305 m asl, 38°34'59"N, 21°38'56"E, 10.iii.2012, Radea, Charou, Papadogiannis, Parmakelis. Nom. IOANNINON: Lake Toumpa, ca 650 m asl, 39°43'31"N, 20°44'53"E, 29.iv.2012, Parmakelis and Triantis; springs of Louros river, ca 285 m asl, 39°25'56"N, 20°50'25"E, 30.iv.2012, Parmakelis and Triantis; spring in Chani Terovo, ca 205 m asl, 39°23'48"N, 20°50'54"E, 30.iv.2012, Parmakelis and Triantis; springs in Ag. Georgios, ca 105 m asl, 39°16'09"N, 20°51'01"E, 30.iv.2012, Parmakelis and Triantis.

###### Remarks.

*Radomaniola* was proposed by Szarowska (2006) as a replacement name for the genus *Orientalina* (Radoman, 1978). According to Radoman (1983), the Balkan species *Orientalina curta* (Küster, 1852) may be divided into a few subspecies on the basis of shell properties and the same author reported the presence of the subspecies *Orientalina curta albanica* Radoman, 1973 from the area of Ioannina. However, the shell dimensions of the specimens we collected from localities of Aitoloakarnania and Ioannina, (shell height: 1.90–3.65 mm, shell width: 1.20–1.95 mm, aperture height: 0.85–1.30 mm, aperture width: 0.70–1.20 mm) are similar to those of *Orientalina curta curta* (Küster, 1852) (shell height: 2.31–3.23 mm, shell width: 1.39–1.93 mm, aperture height: 0.92–1.30 mm, aperture width: 0.84–1.18 mm) reported by Radoman (1983).

Recently, Falniowski et al. (2012) analyzed molecular data from populations of the *Radomaniola*/*Grossuana* group collected from several localities of Greece and other countries of Balkan Peninsula. Two of the Greek localities were “spring in the city centre of Thermos, NE of Trichonida Lake” and “springs of Louros”. In the Bayesian tree based on *COI* sequences provided by Falniowski et al. (2012), the specimens from these localities belong to the genus *Radomaniola* s. stricto. The above authors refer to the specimens from Thermos as “*Trichonia kephalovrissonia*” in the Bayesian tree. This fact indicates that *Trichonia kephalovrissonia* Radoman, 1973 [Schütt (1980) considers this taxon as a synonym of *Semisalsa steindachneri* (Westerlund, 1902) (=*Heleobia (Semisalsa) steindachneri* (Westerlund, 1902)] should be assigned to *Radomaniola kephalovrissonia* (Radoman, 1973). In the Bayesian tree mentioned above, the specimens from Louros seem to be close to *Radomaniola montana* (Radoman, 1973) and rather far from *Radomaniola curta curta* (Küster, 1852) and other *Radomaniola curta* (Küster, 1852) subspecies.

Despite the fact that our sampling localities (Fig. 1) are situated very close to those of Falniowski et al. (2012), we consider that all specimens collected from Aitoloakarnania and Ioannina belong to *Radomaniola curta* as long as only their morphological and anatomical characters have been examined and no molecular data are available.

#### Genus *Trichonia* Radoman, 1973

##### Trichonia trichonica

Radoman, 1973

http://species-id.net/wiki/Trichonia_trichonica

Figure 3

###### New records.

Nom. AITOLOAKARNANIAS: Lake Trichonis, N shore close to Dougri, ca 15 m asl, 38°36'01"N, 21°34'10"E, three specimens on leaves and stems of *Myriophyllum* sp. and *Potamogeton* sp., depth 2-4 m, 09.iii.2012, Radea, Charou, Papadogiannis, Parmakelis.

###### Remarks.

*Trichonia trichonica* was described by Radoman (1973) from Lake Trichonis, by the NE rocky bank near Myrtia but Schütt (1980) states that the species had previously been found in the sub-littoral zone of the stony southern shore of this lake. It seems that the species was found alive and collected from Lake Trichonis for the last time in 1985 (Szarowska 2006). Albrecht et al. (2006, 2009) referred that they did not find alive specimens of this species in Lake Trichonis in 2005, 2007 and 2008. However, a few specimens of *Trichonia trichonica* were discovered close to the new locality by Radea in 2009.

Frogley and Preece (2007) found that *Trichonia trichonica* lives on stones and aquatic vegetation at the mouth of Krya’s spring discharging at the northern bank of another ancient Greek lake, Lake Pamvotis, Ioannina, Ipirus. The fieldwork of the latter authors was carried out in 1994, 1998 and 2005. Recently, Szarowska and Falniowski (2011b) reported that in 2003 there was no trace of the spring on the northern bank of Lake Pamvotis. During our sampling in Lake Pamvotis, we were not able to relocate the species and, additionally, we ascertained that the Krya’s spring was destroyed since a water intake was built on it.

## Discussion

Among other findings of this study, we found *Graecoanatolica vegorriticola* in two new localities quite distant from all the known localities of this taxon. In our effort to properly assign these populations to the species they belong to, we realized that the morphological and anatomical studies were not conclusive. To overcome this issue, *COI* sequence data were used to compare the *Graecoanatolica vegorriticola* specimens collected from the type locality of the species, with those of the newly located southern populations. The level of *COI* sequence divergence (1.7%) between the population from the type locality and those from the new localities is well within the range separating conspecific populations of Hydrobiidae genera, e.g. *Pyrgulopsis* 0–3.44% (Hurt 2004), *Grossuana* 3.4% (Szarowska et al. 2007), *Daphniola* 1.3–2.7% (Falniowski et al. 2007), *Isimerope* 3% (Radea et al. 2013) and other truncatelloidean genera e.g. *Austropyrgus* 3–5% (Perez et al. 2005). Consequently, the above populations are conspecific and belong to *Graecoanatolica vegorriticola*. The genus *Graecoanatolica* is distributed in the Balkans (two species) and in Turkey [eight species (Radoman 1983, 1985, Kebapçi et al. 2012)]. Radoman (1985) refers that the disjunct distribution of the genus could be an evidence of a paleohydrogeographical link between Anatolia and the Balkans. However, this connection could not be verified by molecular and anatomical data as the Balkan species were reported to have gone extinct (Kebapçi et al. 2012). The discovery of fresh specimens of *Graecoanatolica vegorriticola* in three localities of Greece eliminates this obstacle, and the alledged paleohydrogeographical link hypothesis can now be evaluated, provided that sequence data from the Turkish species become available.

Due to insufficient morphological and anatomical differentiation, the nominal subspecies *Pseudamnicola macrostoma macrostoma* (Küster, 1853) and *Pseudamnicola macrostoma negropontina* cannot be discriminated. However, Szarowska et al. (2006) supported that these taxa should be considered as distinct species. This claim was evaluated in the light of the generated sequence data from the newly collected specimens. The sequence divergence separating *Pseudamnicola macrostoma macrostoma* and *Pseudamnicola macrostoma negropontina* is 5.8% and this level of sequence divergence falls within the range 3.7–7.0% [Szarowska and Falniowski (2011), table 5 and p. 126] that separates Greek *Pseudamnicola* species. Therefore, it can be claimed that based on the *COI* sequence divergence levels, additional data exist to support the view that, despite the morphological and anatomical similarity between the two taxa, a species statusshould be assignedto *Pseudamnicola macrostoma negropontina*, as Szarowska et al. (2006) suggested.

During the field survey undertaken for this study, several threatened taxa, such as *Graecoanatolica vegorriticola*, *Pseudamnicola negropontina*, *Pseudamnicola pieperi*, *Pseudobithynia eubooensis* and *Pseudoislamia balcanica*, were recorded from new localities. *Trichonia trichonica*, which has been considered extinct from its type locality for the last twenty eight years (Albrecht et al. 2006), was re-discovered, whereas the presence of *Daphniola exigua*, *Graecoanatolica vegorriticola*, *Marstoniopsis graeca*, *Pseudamnicola pieperi* and *Pseudobithynia trichonis* in their type localities was verified. These findings combined with the recent discovery of the new endemic genus and species, *Isimeropesemele* (Radea et al. 2013), besides confirming the crucial role of the Greek freshwater systems in shaping Europe’s freshwater biodiversity (Glöer and Maassen 2009, Glöer et al. 2010), they also highlight two major issues, a) the Greek freshwater systems have yet a lot to offer to this diversity if comprehensively studied, and b) the IUCN status of some Greek endemic hydrobioids needs to be updated. Towards the latter issue we support that the new data derived from this survey allow us to propose transfers between categories for some species included in the IUCN Red List Threatened Species (2012) ver.3.1 (Table 2). These transfer proposals are based on the newly acquired knowledge regarding the distributional ranges of the species (*Graecoanatolica vegorriticola*, *Pseudoislamia balcanica* and *Trichonia trichonica*) as well as to the elucidation of the standing taxonomic confusion of certain species i.e. *newly split* (*Pseudamnicola negropontina*) and *newly described* (*Isimerope semele*). We followed the IUCN guidelines regarding the definition of the term “location”: “a geographically or ecologically distinct area in which a single event (e.g. pollution) will soon affect all individuals of the taxon present” (IUCN 2011).

*Graecoanatolica vegorriticola*. Our findings indicate that none of the criteria of the category Critically Endangered are met since the extent of occurrence (EOO) and the area of occupancy (AOO) become >100 km^2^ and >10 km^2^, respectively. Therefore, this species may be down-listed to the category Endangered if the criteria of the category Critically Endangered continue to not be met for the next five years. Additionally, (a) and b(iii) are met because the number of locations is ≤ 5 and a continuing decline is observed in the quality of the habitat, respectively; c(iv) is also met because extreme fluctuation in the number of mature individuals has been recorded (Hauffe et al. 2011).

**Table 2.** Scale of endemism of the hydrobioids collected and the suggested transfers between IUCN Red List Categories (with bold our sampling localities).

	Scale of Endemism	IUCN Red List Category (2012) ver. 3.1	Transfers
Family Amnicolidae			
*Marstoniopsis graeca*	E_**PELLA** (**LAKE VEGORRITIS**)_	Critically Endangered B1ab(i,iii)	-
Family Bythinellidae			
*Bythinella charpentieri*, *Bythinella* cf. *charpentieri*	E_ATTIKI+EVVOIA+PARNASSOS Mt._<br/> _**EVVOIA+VOIOTIA**_	Least Concern	-
Family Bithyniidae			
*Pseudobithynia euboeensis*	E_**EVVOIA** (**SPRING**)_	Critically Endangered B2ab(iii)	-
*Pseudobithynia trichonis*	E_**AITOLOAKARNANIA** (**LAKE TRICHONIS**+LAKE LYSIMACHEIA)_	Endangered B1ab(iii)	-
Family Hydrobiidae			
*Daphniola exigua*	E_**THESSALIA** (**AG. PARASKEVI SPRING**+DAPHNI SPRING)_	Endangered B2ab(iii)	-
*Graecoanatolica vegorriticola*	E_**PELLA** (**LAKE VEGORRITIS**+LAKE PETRON)+**VOIOTIA** (**SPRINGS**)_	Critically Endangered B1ab(i,iii,iv)c(iv)+2ab(i,iii,iv)c(iv)	Endangered B1ab(iii)c(iv)
*Isimerope semele*	E_**ARGOLIDA** (**SPRINGS**)+**ARKADIA** (**RIVER**+SPRING)_	Not Evaluated	Endangered B1ab(iii)
*Pseudamnicola negropontina*	E_**EVVOIA** (**SPRING**)_	Not Evaluated	Critically Endangered B2ab(ii, iii)
*Pseudamnicola pieperi*	E_**KARPATHOS** (**SPRINGS**)_	Vulnerable D2	-
*Pseudoislamia balcanica*	E_**AITOLOAKARNANIA** (**LAKE TRICHONIS+SPRING**)_	Critically Endangered B1ab(iii)	Endangered B1ab(iii)
*Radomaniola curta*, *Radomaniola* cf. *curta*	E_ALBANIA+GREECE_<br/> _**AITOLOAKARNANIA+IOANNINA**_	Least Concern	-
*Trichonia trichonica*	E_**AITOLOAKARNANIA** (**LAKE TRICHONIS**)_	Critically Endangered B2ab(i,iii)	Critically Endangered B1ab(iii)

*Pseudoislamia balcanica*. The discovery of this species in a new locality increases the extent of its occurrence (EOO), which becomes >100 km^2^. Therefore, *Pseudoislamia balcanica* may be down- listed to the category Endangered if it continues to thrive in other locality(ies), apart from Lake Trichonis, at least for the next five years. Additionally, (a) and b(iii) are met because the number of locations is ≤ 5 and a continuing decline is observed in the quality of the habitat, respectively.

*Trichonia trichonica*. We re-discovered this species in Lake Trichonis. On the contrary, no single specimen or even empty shells were recorded in Krya’s spring (Lake Pamvotis) due to the water intake built on it. Therefore, the criterion B1, the extent of occurrence (EOO) <100 km^2^, is met for *Trichonia trichonica*. Additionally, (a) and b(iii) are met because the number of locations is 1 and a continuing decline is observed in the quality of the habitat, respectively.

*Pseudamnicola negropontina* has not yet been evaluated because it is now elevated to species level (*newly split*). The area of occupancy of this species is < 10 km^2^ and, consequently, the criterion B2 is met. Additionally, (a) and b(ii,iii) are met because the number of locations is 1 and a continuing decline is observed in the area of occupancy and the quality of the habitat, respectively.

*Isimerope semele* has not yet been evaluated because it is a newly described species (*newly described*). The extent of occurrence of this species is 100 km^2^<EOO<5000 km^2^ and therefore the criterion B1 for the category Endangered is met. Additionally, (a) and b(iii) are met because the current distribution of the species is severely fragmented and a continuing decline is observed in the quality of the habitat, respectively.

During the field survey we ascertained that many of the “hydrobioid” localities in Greece are heavily influenced by various human activities such as tourism, agriculture, livestock, industry, housing development and forestry. Thus, a decline or even loss of local freshwater gastropods is expected and, in some cases, it has already been reported (Ryan and Griffiths 2001, Szarowska and Falniowski 2004, Albrecht et al. 2006, Regnier et al. 2009).

Against the loss of hydrobioids due to the declining number of suitable habitats, a taxonomically accurate record of taxa, especially for those thriving in springs and spring brooks (crenobionts), will contribute significantly in assigning high conservation priorities (Arconada and Ramos 2001, Haase 2003). Without serious and effective intervention it is only a matter of time before the vast majority of the unprotected hydrobioids we recorded in this study become extinct.

## Supplementary Material

XML Treatment for Marstoniopsis graeca

XML Treatment for Bythinella cf. charpentieri

XML Treatment for Pseudobithynia euboeensis

XML Treatment for Pseudobithynia trichonis

XML Treatment for Daphniola exigua

XML Treatment for Graecoanatolica vegorriticola

XML Treatment for Isimerope semele

XML Treatment for Pseudamnicola negropontina

XML Treatment for Pseudamnicola (Pseudamnicola) pieperi

XML Treatment for Pseudoislamia balcanica

XML Treatment for Radomaniola cf. curta

XML Treatment for Trichonia trichonica
